# Characterization of Chemical Degradation in Lithium-Ion Batteries Using Secondary Ion Mass Spectrometry (SIMS) and Hard X‑ray Photoelectron Spectroscopy (HAXPES)

**DOI:** 10.1021/acsomega.5c08521

**Published:** 2025-11-19

**Authors:** Abdulrhman H. Alsaedi, Ben F. Spencer, Sadia Sheraz, Alex S. Walton, Nicholas P. Lockyer

**Affiliations:** 1 Department of Chemistry and Photon Science Institute, 5292 The University of Manchester, Manchester M13 9PL, United Kingdom; 2 Department of Materials and Henry Royce Institute, 5292 The University of Manchester, Manchester M13 9PL, United Kingdom; 3 Department of Chemistry, Faculty of Science, 441424 University of Jeddah, Jeddah 23218, Saudi Arabia

## Abstract

LiNi_*x*_Mn_*y*_Co_*z*_O_2_ layered oxide cathodes are widely used in lithium-ion batteries, with LiNi_1/3_Mn_1/3_Co_1/3_O_2_ being the most commercially popular. Increasing the nickel ratio enhances the battery capacity and energy density but also quickens electrolyte degradation and capacity fade. This work presents a comparative analysis of the noncharged and single-charged states of NMC111, NMC532, and NMC811 cathodes using SIMS and HAXPES. The results reveal that electrolyte degradation initiates before applying a voltage bias and increases during the first charge, correlating with both increasing nickel content and charging process. Further comparison of NMC811 cathodes cycled in two different electrolytes (E1 and E2) across different electrochemical states reveals distinct cathode electrolyte interphase (CEI) evolution. In E1, the CEI layer reaches maximum thickness after a single charge and subsequently decreases upon discharge and at the end of life. In contrast, in the E2 electrolyte, the CEI layer continues to grow progressively throughout cycling. SIMS depth profiling at the discharged and end-of-life states shows a dual-layer CEI structure in both electrolytes with deeper penetration and denser accumulation observed in the E2 electrolyte system. Consistent with previous hypotheses, this study demonstrates that the CEI thickness is affected by both the nickel content and electrolyte reactivity.

## Graphical abstract

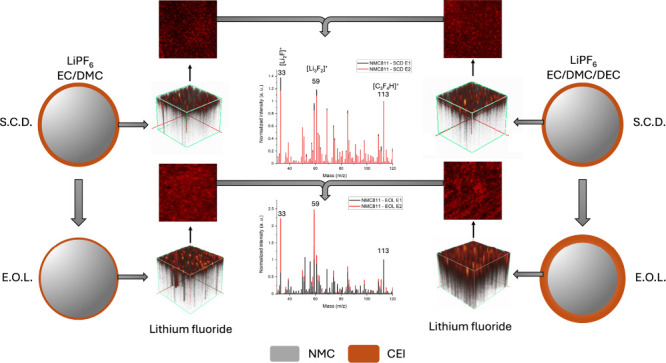

## 1 Introduction

The growing demand for high-energy density, long cycle life, and safe batteries has driven considerable improvements in battery technology. Lithium-ion batteries (LIBs) have been integrated into applications ranging from portable electronics to electric vehicles. Their popularity is due to the advantages of high-energy capacity, high potential, minimal memory effect, extended cycle life, and effective operation across a wide temperature range.^1,2^ These features make LIBs a preferred choice in modern energy storage systems.

To further increase cell voltage and energy density, Goodenough and co-workers pioneered the use of oxide cathodes, aiming to lower the redox energy of the cathode.^3^ This work identified three main types of oxide cathodes: layered oxide,^4,5^ spinel oxide,^6^ and polyanion oxide.^7^ Among these, Li_*x*_CoO_2_ was initially used, exhibiting an open-circuit voltage almost double that of the sulfide-based Li_*x*_TiS_2_ cathode, which has a voltage of below 2.5 V.^4^ However, Li_*x*_CoO_2_ faces the challenge of oxygen release from the lattice at a high charge state, which leads to performance degradation.

The high cost of cobalt and the need for increasing the cell capacity have led to the investigation of alternative cathode materials, such as LiNi_*x*_Mn_*y*_Co_*z*_O_2_ (NMC), with *x* + *y* + *z* = 1. Various NMC cathodes with different metal ratios (e.g., NMC111, NMC532, NMC622, and NMC811) have been explored to optimize performance and stability. NMC cathode materials possess higher thermal stability compared to their LiMO_2_ (M = Ni, Mn, or Co) counterparts. In addition, high-energy-density systems are achievable using a nickel-rich NMC cathode like NMC811. However, NMC811 suffers from limited cycle life due to severe interactions with the electrolyte at the electrode surface, resulting in the formation of the cathode electrolyte interphase (CEI).^8,9^ The CEI layer is formed due to the narrow stability window of organic electrolytes commonly used in LIBs.^2,8,10,11^ Furthermore, although NMC811 cathodes are already commercialized, challenges such as metal dissolution and cation mixing affect battery performance and longevity. These challenges underscore the need for further stabilization strategies to enhance stability during cycling and extend the operational lifespan.^12^

While the formation of the CEI layer is unavoidable, its nature considerably impacts the battery performance. An unstable or thick CEI can impede lithium-ion transport and contribute to capacity loss, particularly in nickel-rich cathodes. However, a stable CEI can act as a protective barrier that restricts further electrolyte decomposition and stabilizes the interface, thereby enhancing battery longevity.^13^ The challenge lies in adjusting both morphology and chemical composition of the CEI layer.^8^ Despite various efforts to enhance the stability of this layer, both in situ through the additives^14^ and ex-situ by electrode surface coatings,^15^ current strategies remain inadequate. Although the CEI layer is the most crucial aspect of LIBs, it is still not well-understood, especially regarding its spontaneous and electrochemical formation of the electrode surface.

In this study, we use time-of-flight secondary ion mass spectrometry (ToF-SIMS) and Hard X-ray Photoelectron Spectroscopy (HAXPES) to examine the passivation layers formed on NMC cathode electrodes. ToF-SIMS, with its nanometer surface sensitivity and high chemical selectivity, provides a detailed surface composition analysis and depth profiling capabilities beyond the information depth of HAXPES. Meanwhile, HAXPES offers quantitative insights into the passivation layer’s depth and composition. By combining these complementary techniques, we aim to advance our understanding of the CEI structures that underpin LIB stability and performance.

## 2 Experimental Section

### 2.1 Electrode Fabrication

The three types of cathode electrodes were used in this study include LiNi_0.33_Mn_0.33_Co_0.33_O_2_ (NMC111), LiNi_0.5_Mn_0.3_Co_0.2_O_2_ (NMC532), and LiNi_0.8_Mn_0.1_Co_0.1_O_2_ (NMC811). These materials were obtained from Pi-Kem Ltd. (Tamworth, UK). For the cathode electrodes, a mixture of active materials (NMC), carbon additive (Super P), and a binder (poly­(vinylidene fluoride), PVdF) in a ratio of 90:5:5 was coated onto aluminum foil.

The electrodes were cycled against the graphite anode electrode, which was obtained from Cambridge Energy Solutions Ltd. (Cambridge, UK). For the anode electrode, a mixture of the active material and the binder (carboxymethyl cellulose (CMC) and sodium butadiene rubber (SBR)) with a ratio of 93.2:6.8 was coated onto copper foil.

Two electrolyte systems were used in this study. The first, termed E1, consisted of 1 M LiPF_6_ in ethylene carbonate (EC) and dimethyl carbonate (DMC) with a 1:1 ratio. The second, referred to as E2, consisted of 1 M LiPF_6_ in ethylene carbonate (EC), dimethyl carbonate (DMC), and diethyl carbonate (DEC) with a 1:1:1 ratio. Both electrolytes were obtained from Sigma-Aldrich (Gillingham, UK). A Celgard 2325 trilayer separator was used, which was obtained from Cambridge Energy Solutions Ltd. (Cambridge, UK).

The electrochemistry was performed by using CR2032-type coin cells. These batteries were cycled between 2.8 and 4.2 V vs Li+/Li at room temperature. Electrodes exposed to electrolyte for 12 h without electrochemical cycling are referred to as noncharged (N.C.), while those that have been single-charged or single-charged and discharged are referred to as S.C. or S.C.D. electrodes, respectively. Electrodes that have been cycled to their end-of-life (E.O.L.), i.e., a decrease to 80% of their initial capacity, were also analyzed.

### 2.2 Material Characterization

ToF-SIMS analysis was performed using a J105 3D chemical imager (Ionoptika Ltd., Chandler’s Ford, UK).^16^ A 40 keV C_60_^+^ beam was used as primary ions. 3D images from an area of 500 × 500 μm^2^ with 128 × 128 pixels were acquired with a primary ion dose of 4.5 × 10^12^ ion/cm^2^. The pressure of the analysis chamber is ∼10^–8^ mbar.

The HAXPES instrument utilized in this study is a laboratory-based HAXPES instrument (Scienta Omicron Ltd., Uppsala, Sweden). Gallium metal-jet is used as an X-ray source (9.25 keV, 3.57 mA emission at 250 W).^17,18^ Measurements were operated at grazing incidence (approximately normal emission).^17^ Survey spectra were measured using an entrance slit width of 1.5 mm with 500 eV electron energy analyzer pass energy, and core level spectra were taken using 100 eV pass energy, with energy resolutions of 2.0 and 0.6 eV.^17^ High-resolution scans were taken of the carbon, oxygen, and fluorine core levels with a size step of 0.2 eV. The binding energy calibration was performed by referencing the C–H/C-C components of the C 1s peak to 284.8 eV. The pressure of the analysis chamber was maintained at ∼10^–9^ mbar.

### 2.3 Sample Transfer

Cells were disassembled inside an Ar-filled glovebox (MBraun), with oxygen and water level <0.5 ppm. The harvested electrodes were washed with DMC and then transferred to SIMS and HAXPES using a vacuum vessel, which can be directly fitted into the HAXPES load lock. The J105 instrument, equipped with glovebox, allows for Ar-purging and cooled with LN2 cooling to reduce humidity before mounting the sample for SIMS analysis. The relative humidity content inside the glovebox was maintained below 1% during sample transfer.

## 3 Results and Discussion

### 3.1 Comparing NMC Electrode Behavior: Noncharged (N.C.) vs Single-Charged (S.C.) States

The formation of the CEI layer on the electrode begins as soon as it is immersed in the electrolyte, even without applying any bias, and continues to evolve with cycling.^19^ To compare the reactivity of different electrodes, we examined both N.C. and S.C. states for NMC111, NMC532, and NMC811 cycled with the E1 electrolyte. Figure 1 shows the O 1s spectra for both N.C. and S.C. states in panels (a) and (b), with their corresponding atomic percentages in panels (c) and (d), respectively. Similarly, Figure 2 presents F 1s spectra for N.C. and S.C. states panels in (a) and (b), alongside their atomic percentages in panels (c) and (d). Peak assignments for O 1s and F 1s are summarized in Table S1 based on previous studies.^20–22^

**Figure 1:**
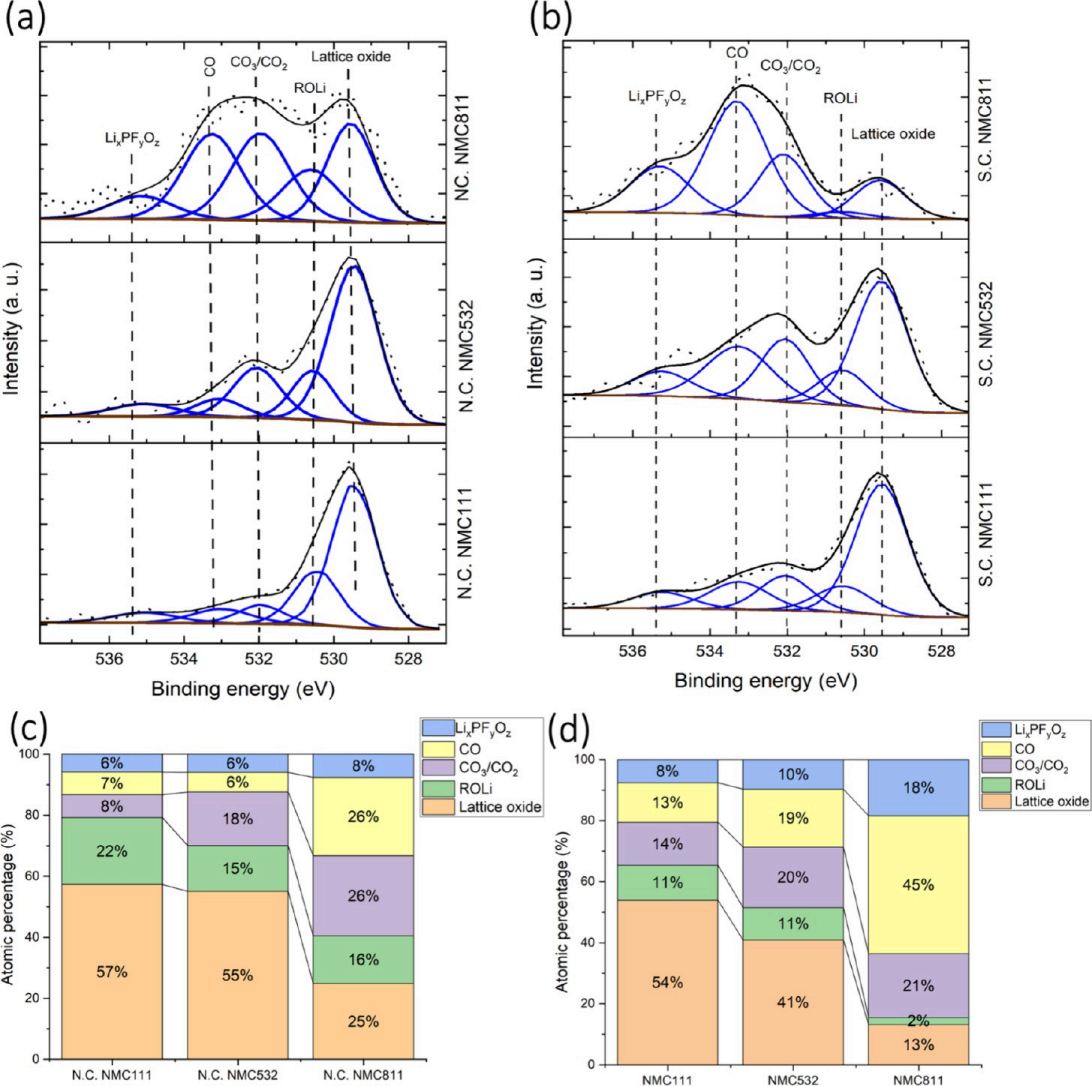
HAXPES O 1s spectra for N.C. states (a) and S.C. states (b) of NMC111, NMC532, and NMC811, with corresponding atomic percentages of O 1s components shown in panels (c) and (d), respectively.

**Figure 2:**
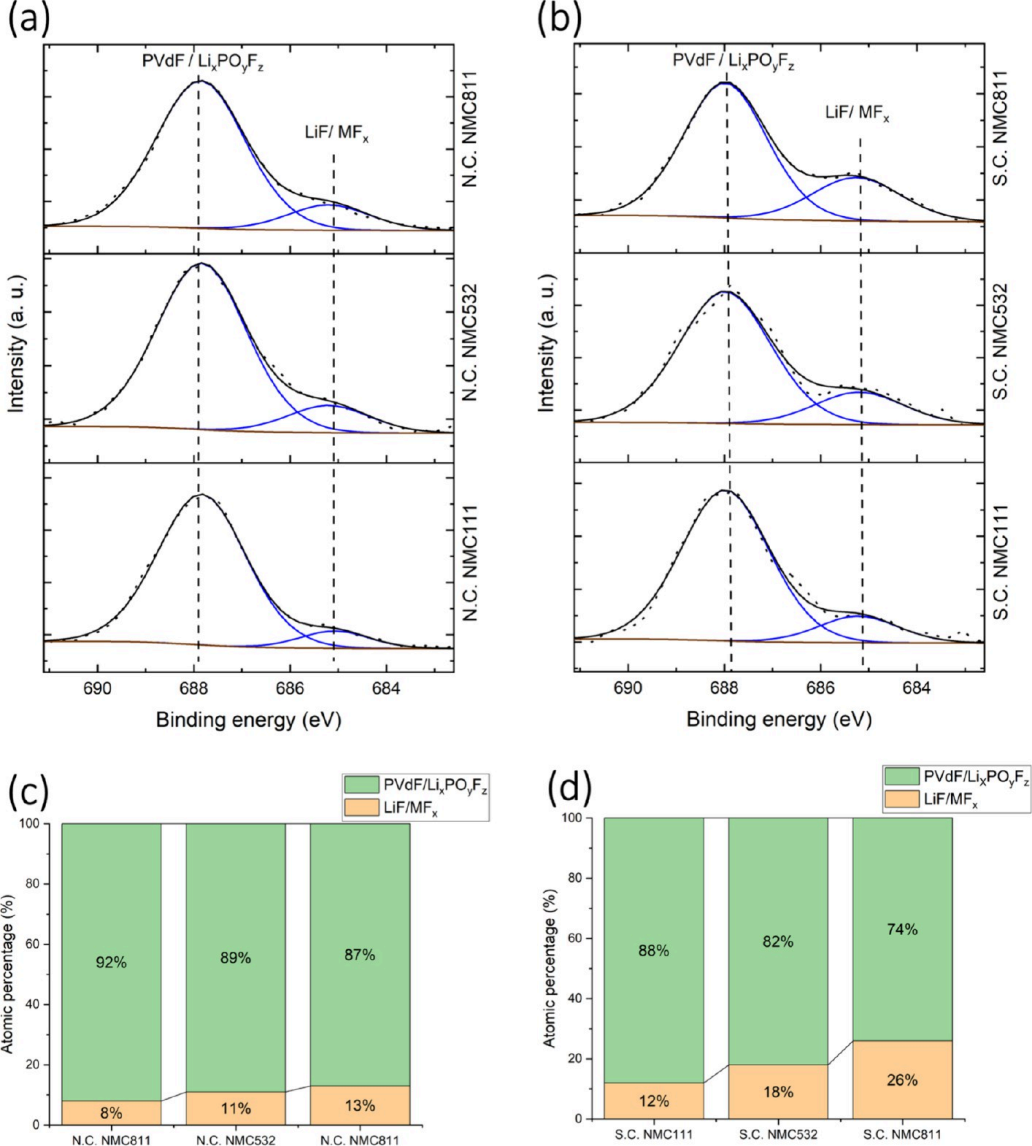
HAXPES F 1s spectra for N.C. states (a) and S.C. states (b) of NMC111, NMC532, and NMC811, with corresponding atomic percentages of F 1s components shown in panels (c) and (d), respectively.

The O 1s spectra were fitted with five peaks corresponding to lattice oxide, ROLi, CO_3_/CO_2_, CO, and Li_*x*_PF_*y*_O_*z*_. HAXPES detected lattice oxide across all samples, indicating that the thickness of the CEI layer is less than the probing depth for HAXPES, allowing signal collection from the entire CEI. The inelastic mean free path (IMFP) of lattice oxide photoelectrons in the NMC111, NMC532, and NMC811 electrodes was estimated using the TPP-2 M formula. At a photoelectron binding energy of approximately 529.5 eV, the IMFP was calculated to be approximately 13 nm, corresponding to an information depth of about 39 nm. These values suggest that HAXPES is effective for studying buried layers, such as the passivation layers.

The presence of CO_3_/CO_2_ and CO peaks indicates electrolyte decomposition, likely producing lithium carbonate from reactions involving lithium compounds, CO_2_, and H_2_O.^22–24^ The Li_*x*_PF_*y*_O_*z*_ peak highlights electrolyte salt degradation, and its detection in the N.C. state reveals spontaneous electrolyte–electrode reactions (Figure 1a).^25^

The intensity of the lattice oxide peak provides valuable insights into CEI layer thickness.^21^ In the N.C. state, lattice oxide intensities are 57, 55, and 25% for NMC111, NMC532, and NMC811, respectively, and these values decrease in the S.C. state to 54, 41, and 13%. This reduction in the lattice oxide atomic percentage across all electrodes in the S.C. state implies an increase in CEI layer thickness during cycling. Additionally, a trend emerges where lattice oxide intensities decrease as nickel content increases in both N.C. and S.C. states, indicating that a higher nickel content correlates with a thicker CEI layer. This is further supported by rise in CO_3_/CO_2_, CO, and Li_*x*_PF_*y*_O_*z*_ components, indicating a more evolved and thicker CEI layer in the S.C. state (Figure 1b). Higher nickel content in the NMC cathode intensifies the catalytic decomposition of carbonate solvents, leading to generation of protic species that accelerate salt decomposition.^26^

Figure 2a,b presents the F 1s spectra, where two distinct peaks were fitted. The shoulder at a lower binding energy (∼685 eV) corresponds to metal fluorides (MF_*x*_) and LiF, while the peak at a higher binding energy (∼687.9 eV) corresponds to PVdF and Li_*x*_PF_*y*_O_*z*_. The formation of LiF and Li_*x*_PF_*y*_O_*z*_ is correlated to electrolyte salt degradation,^21^ while MF_*x*_ components are believed to form due to the attack of acidic species, such as HF (from trace H_2_O impurities).^27^ The detection of these components in the N.C. state again shows spontaneous reactions between the electrolyte and electrode prior to electrochemical cycling.

The corresponding atomic percentages of these components, as shown in Figures 2c,d, reveal that the ∼685 eV peak increases with nickel content and becomes more pronounced in the S.C. state. This trend indicates that CEI layer growth is strongly influenced by the nickel content and evolves during cycling. This suggests that salt degradation is more significant in nickel-rich electrodes, further underscoring nickel’s role in enhancing electrolyte decomposition within the CEI layer.

Lithium fluoride can serve as an indicator of CEI layer thickness due to its prevalence in the CEI layer. Figure 3 shows the primary ion dose required to remove lithium fluoride from the N.C. and S.C. states of various NMC electrodes in the SIMS experiment. The etch dose is defined as the point where the normalized intensity of the [Li_2_F]^+^ ion signal decreases to 0.5. A higher dose is consistently observed in the S.C. state, indicating that CEI formation increases under charged conditions. Notably, for both the N.C. and S.C. states, nickel-rich electrodes require a higher dose to remove lithium fluoride, highlighting nickel’s influence on CEI thickness and electrode reactivity.

**Figure 3:**
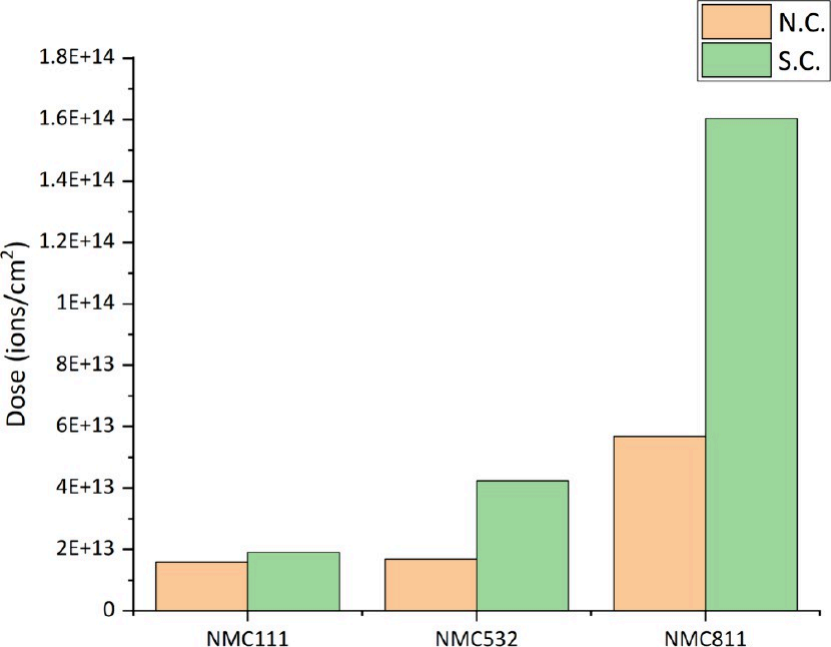
Primary ion dose required to remove the lithium fluoride component from the electrode surface in the N.C. and S.C. states of NMC111, NMC532, and NMC811 in SIMS measurement.

The combined evidence from HAXPES and ToF-SIMS confirms that CEI thickness increases with higher nickel content, reflecting the high reactivity of nickel-rich materials. Additionally, the presence of the CEI components in the N.C. state, followed by further thickening in the S.C. state, suggests that the CEI layer formation initiates spontaneously, which is then driven by electrochemical processes during charging.

### 3.2 CEI Layer Formed on the NMC811 Electrode with Different Electrolytes

To investigate the impact of electrolyte composition on the formation and evolution of the CEI layer in NMC811 electrodes, cycling experiments were conducted using two different electrolytes, E1 and E2 electrolytes. Electrodes were examined at various states of charge.

Figure 4a,c provides a detailed examination of the O 1s spectra for NMC811 electrodes cycled with E1 and E2 electrolytes, with corresponding atomic percentages shown in Figure 4b,d. In the pristine NMC811 powder, four O 1s peaks are observed, corresponding to lattice oxide, ROLi, CO_3_/CO_2_, and CO (Figure S1), with the lattice oxide peak being prominent. Upon cycling, an additional peak emerges at ∼535.4 eV, attributed to Li_*x*_PF_*y*_O_*z*_ species (Figure 4a,c). This feature suggests the formation of phosphate-containing species during cycling, likely originating from electrolyte decomposition and subsequent reactions with the electrode surface.

**Figure 4:**
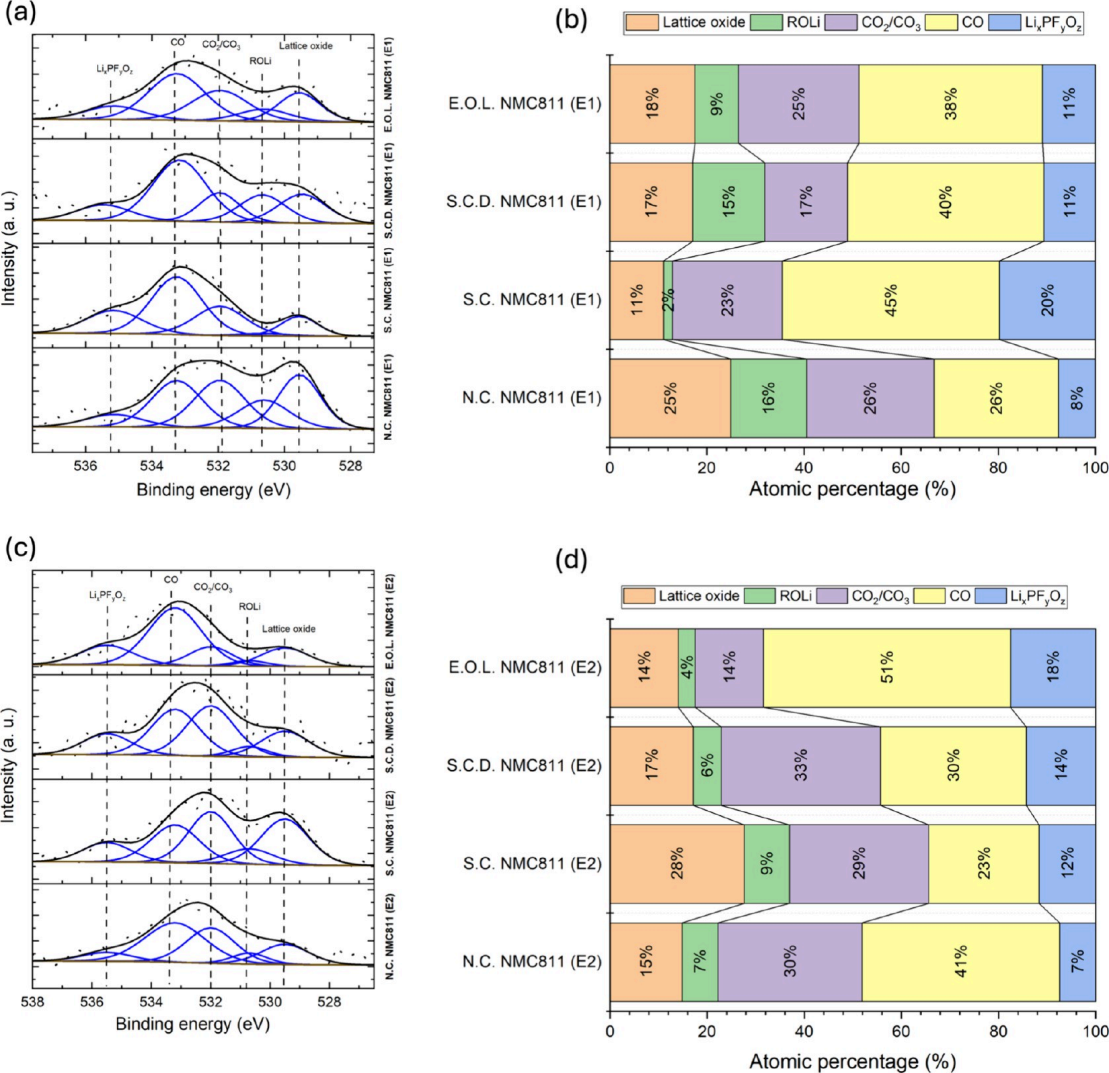
HAXPES O 1s spectra of NMC811 electrodes cycled with (a) E1 and (c) E2 electrolytes at different states of charge. The corresponding atomic percentages of the O 1s components are shown in panels (b) and (d), respectively.

For electrode cycled with the E1 electrolyte, the lattice oxide, an indicator for CEI thickness, shows a decline from 25% in the N.C. state to a minimum of 11% in the S.C. state, followed by a slight increase to 17 and 18% in the S.C.D. and E.O.L. states, respectively. These changes are likely due to the surface reconstruction and progressive formation of CEI components, such as lithium fluoride, transition metal fluorides, and oxides. This suggests that CEI layer initiates spontaneously in the N.C. state but becomes increasingly electrochemically driven during cycling.^28^

In contrast, electrodes cycled in E2 present a different trend. The lattice oxide intensity initially rises from 15% in the N.C. state to 28% in the S.C. state before decreasing to 17 and 14% in the S.C.D. and E.O.L. states, respectively. This behavior implies that the E2 electrolyte leads to a different CEI growth mechanism, possibly involving delayed or less initial interfacial reactions compared to E1.

The evolution of the CO and Li_*x*_PF_*y*_O_*z*_ species further highlights this contrast. In the E1 system, both species peak in the S.C. state and then decrease in latter stages. The initial increase in these species during the early stages of cycling suggests that they are generated as a result of electrolyte decomposition and subsequent consumed or transformed as the cell continues to cycle. This trend reflects surface composition changes over the battery cycle, likely due to passivation layer dissolution in the electrolyte during cycling.^29^ The reduction in quantity of CO during cycling may indicate decomposition of organic species.^30^

Conversely, in the E2 system, Li_*x*_PF_*y*_O_*z*_ species increase progressively from 7% in the N.C. state to a higher level throughout cycling. Meanwhile, the intensity of the CO peak, which represents carbonate species, decreases initially in the S.C. state but then increases significantly in the S.C.D. and E.O.L. states, reaching 51% at the E.O.L. state. This suggests that carbonate formation continued during cycling, potentially due to electrolyte decomposition.

Figure 5a,c displays the F 1s spectra for NMC811 electrodes cycled with E1 and E2 electrolytes, respectively, with corresponding atomic percentages in Figure 5b,d. The F 1s spectra are fitted with two main peaks: a lower binding energy shoulder corresponding to LiF and other metal fluorides (MF_*x*_) and a higher binding energy assigned to PVdF and Li_*x*_PO_*y*_F_*z*_.

**Figure 5:**
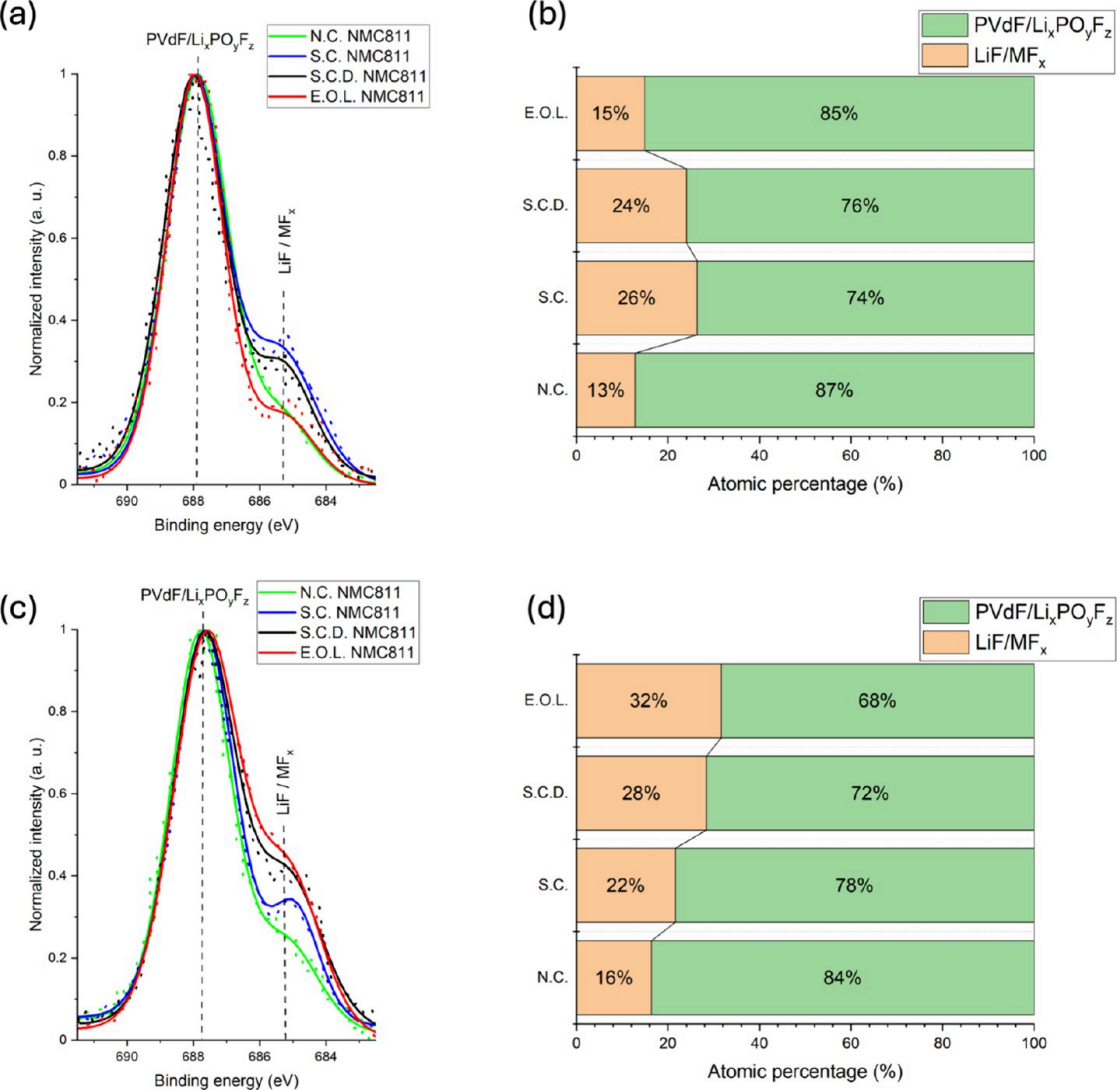
HAXPES F 1s spectra of NMC811 electrodes cycled with (a) E1 and (c) E2 electrolytes at different states of charge. The corresponding atomic percentages of the O 1s components are shown in panels (b) and (d), respectively.

For the E1 electrolyte, the intensity of the LiF and MF_*x*_ shoulder increases from 13% in the N.C. state to 26% in the S.C. state, followed by decreases to 24 and 15% in the S.C.D. and E.O.L. states, respectively. This trend suggests that the formation of LiF and MF_*x*_ is more pronounced during the initial cycling stage but diminishes with cycling, possibly due to the changes of the surface chemistry or consumption of available fluorine-containing species. The decrease in LiF in the E.O.L. state may suggest LiF detachment during cycling as a result of CEI decomposition.^31^

For electrodes cycled with the E2 electrolyte, the CEI composition shows a progressive increase of LiF and MF_*x*_ components with cycling. Starting at 16% in the N.C. states, this value rises to 22% in the S.C. state and continues to increase to 28 and 32% in the S.C.D. and E.O.L. states, respectively. This continuous increase indicates that the E2 electrolyte promotes ongoing formation of LiF and metal fluoride throughout the cycling process.

These findings underscore that CEI formation on NMC811 is strongly electrolyte dependent. While E1 stimulates fast CEI development, followed by partial decomposition, E2 leads to ongoing interfacial reactions and accumulation of degradation components throughout the cycling process.

The C 1s spectra of the NMC811 electrodes, shown in figure S2 for the E1 and E2 electrolytes, exhibit similar peak shapes throughout all cycling states. The spectra are fitted with five peaks: the C–C/C–H peak at ∼248.8 eV; two peaks at ∼286.6 and ∼290.8 eV, corresponding to the polymeric binder PVdF (CH_2_–CF_2_); and peaks at 287.8 and 289.2 eV, attributed to CO and C=O groups, respectively. The similarity in the C 1s spectra across different states indicates that the organic components and the PVdF binder remain relatively stable during cycling. However, slight variations in the intensity of C–O and C=O peaks suggest changes in the surface chemistry of the electrode during cycling. These minor changes may reflect a minor organic component accumulating on the electrode surface.

To investigate the chemical composition across the CEI layer, ToF-SIMS depth profiling was performed. Figure 6 shows the positive ion ToF-SIMS surface spectra of NMC811 electrodes at the S.C.D. and E.O.L. states after cycling in the E1 and E2 electrolytes. These spectra were normalized to the peak at *m*/*z* 113, assigned to [C_3_F_4_H]^+^, a fragment associated with the PVdF binder. Key peaks include *m*/*z* 33 and 59, relating to [Li_2_F]^+^ and [Li_3_F_2_]^+^, respectively; both are associated with lithium fluoride. At the S.C.D. state, the relative intensities of these peaks are relatively similar for electrodes cycled in both electrolytes. However, at the E.O.L. state, the lithium fluoride peaks are three times more intense for the electrode cycled in the E2 electrolyte, suggesting extensive LiF accumulation and CEI growth.

**Figure 6:**
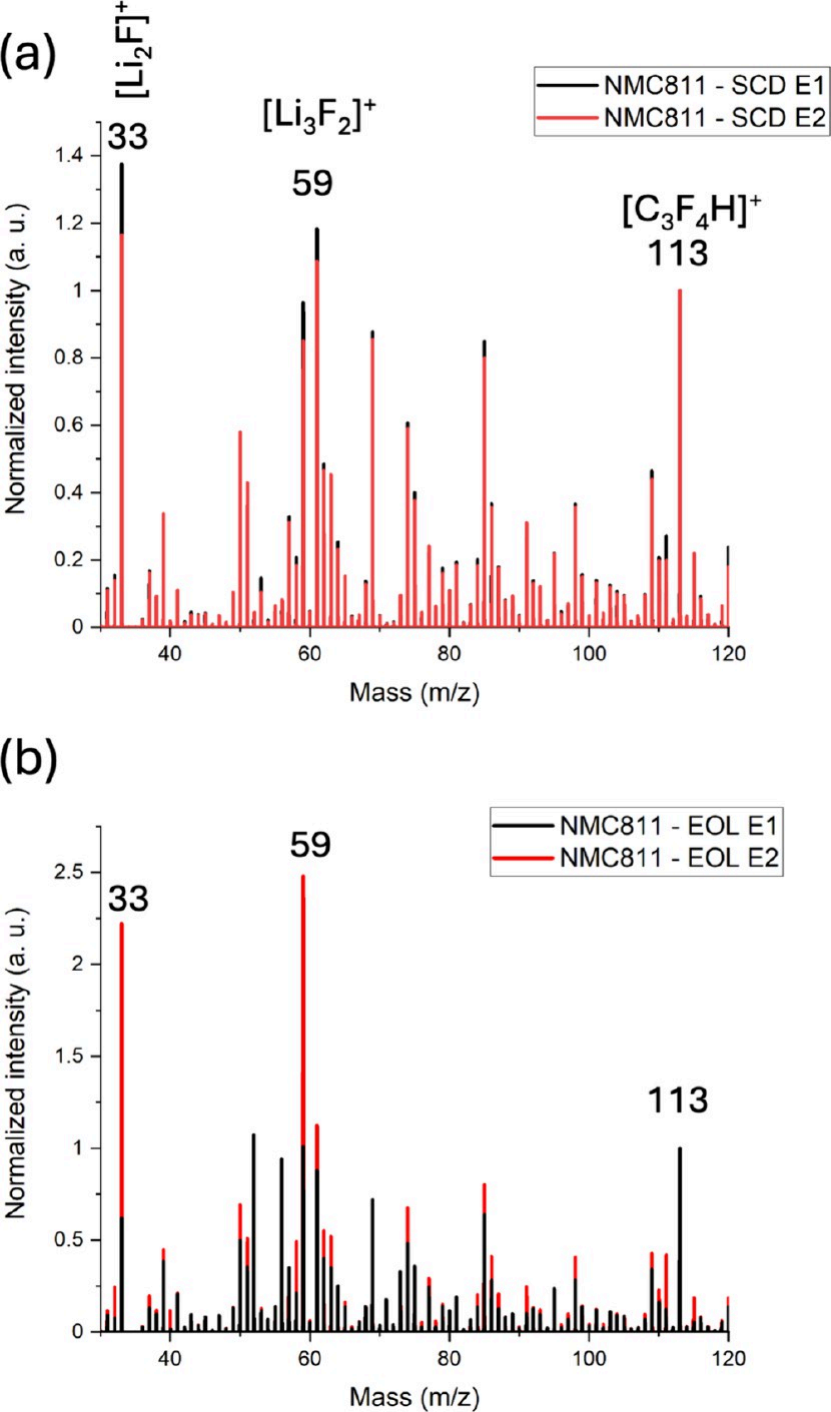
ToF-SIMS surface spectra of NMC811 electrodes at the (a) S.C.D. and (b) E.O.L. states after cycling in the E1 (black) and E2 (red) electrolytes.

Figures 7 and 8 present ToF-SIMS depth profiles of key ion fragments as a function of ion dose for the NMC811 electrode at the S.C.D. and E.O.L. states after cycling with E1 and E2 electrolytes, respectively, with Table 1 listing detected ions, with their mass and mass accuracy detailed in Tables S2 and S3. In both figures, panels (a) and (b) display the positive ion depth profiles, while panels (c) and (d) illustrate the negative ion depth profile for electrodes cycled in E1 and E2, respectively.

**Table 1:** List of Positive and Negative Ions Detected in the ToF-SIMS Analysis along with Their Assumed Assignments

positive ions	assignment	negative ion	assignment
[C_3_F_4_H]^+^	PVdF binder	**[LiF**_**2**_ **]** ^**–**^	lithium fluoride
[Li_2_F]^+^	lithium fluoride	**[PO**_**3**_ **]** **^–^**	phosphorus oxide
[Li_3_O]^+^	lithium oxide	**[PF**_**2**_ **O**_**2**_ **]** **^–^**	fluorophosphate
[Li_3_CO_3_]^+^	lithium carbonate	**[MnF**_**3**_ **]** ^**–**^ **/[NiF**_**3**_ **]** ^**–**^ **/[CoF**_**3**_ **]** ^**–**^	metal fluoride
[Li_a_M_b_O_c_]^+^	lithium metal oxide	**[MnO**_**3**_ **]** ^**–**^ **/[NiO**_**2**_ **]** ^**–**^ **/[CoO**_**2**_ **]** ^**–**^	metal oxide
[Mn]^+^/[Co]^+^/[Ni]^+^	metal		

**Figure 7:**
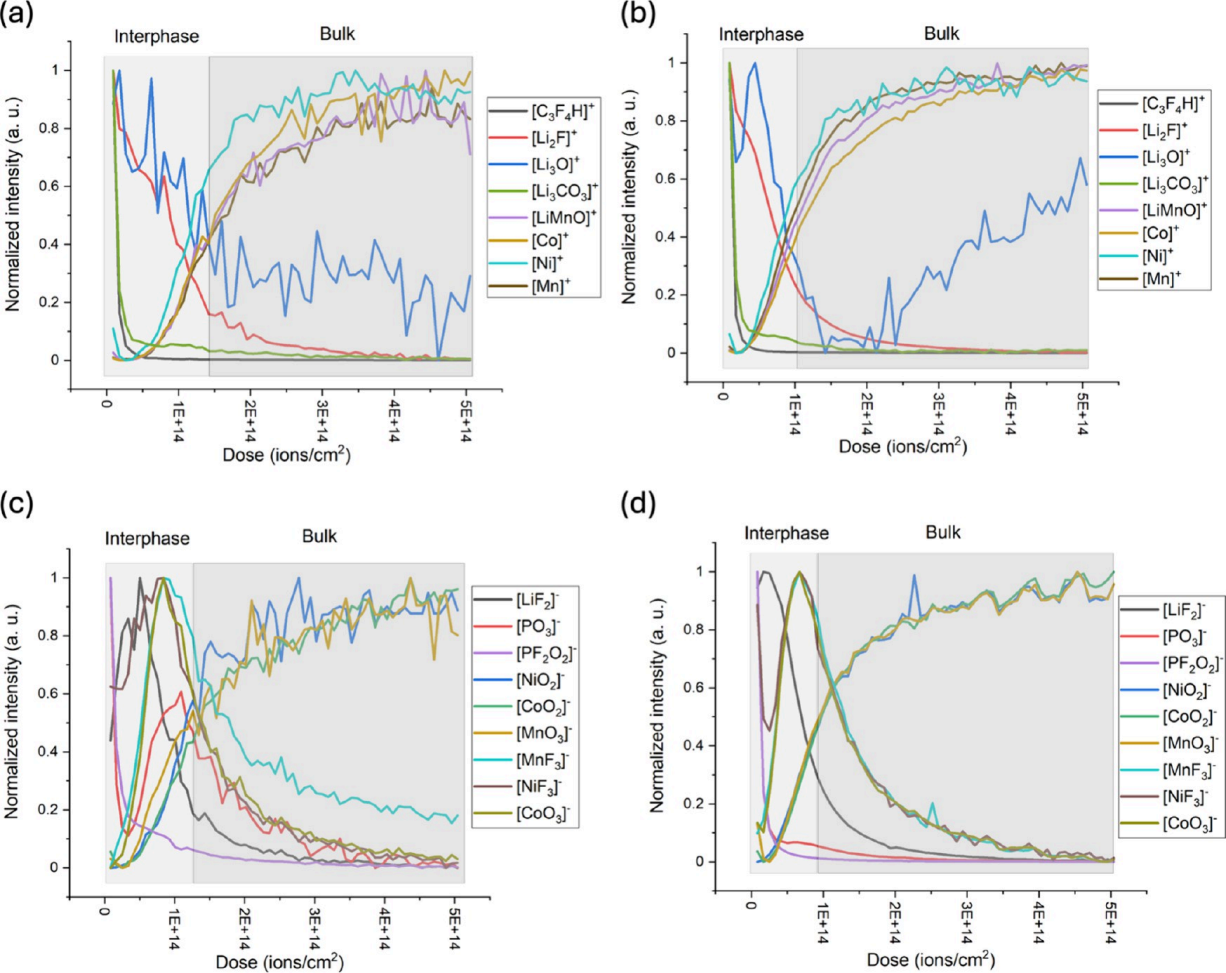
ToF-SIMS depth profiles of NMC811 electrodes in the S.C.D. state. Panels (a) and (c) show the depth profiles acquired in positive and negative ion modes, respectively, for electrodes cycled with electrolyte E1. Panels (b) and (d) show the corresponding profiles for electrodes cycled with electrolyte E2.

**Figure 8:**
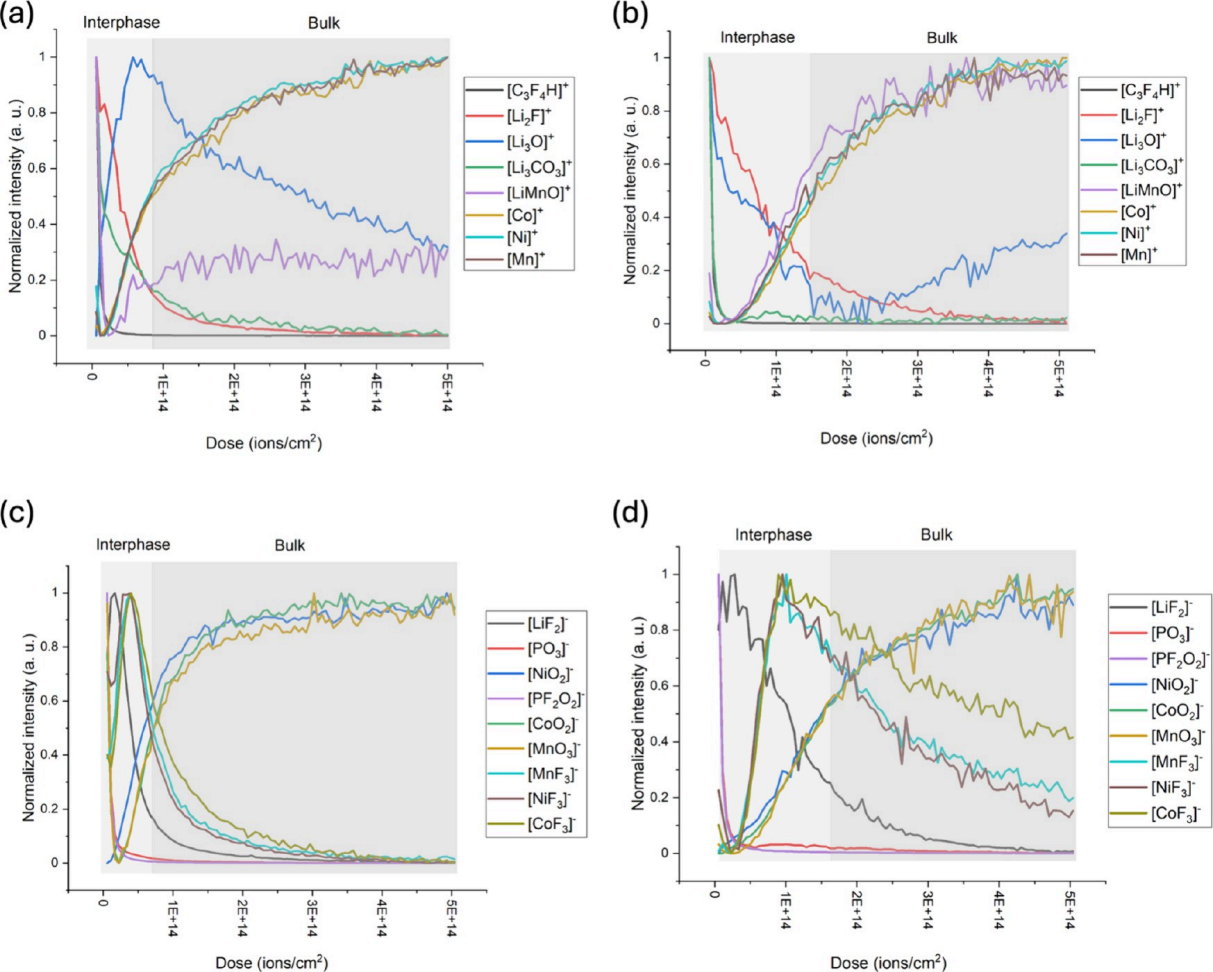
ToF-SIMS depth profiles of NMC811 electrodes in the E.O.L. state. Panels (a) and (c) show the depth profiles acquired in positive and negative ion modes, respectively, for electrodes cycled with electrolyte E1. Panels (b) and (d) show the corresponding profiles for electrodes cycled with electrolyte E2.

The interphase thickness, as labeled in the figures, was determined based on the ion dose at which the normalized intensity of metal ions (positive polarity) and metal oxides (negative polarity) reaches 0.5. At the S.C.D. state, the interphase on the NMC811 electrode surface cycled with the E1 electrolyte is relatively thicker than that formed with the E2 electrolyte (Figure 7a,b). However, at the E.O.L. state, the interphase is thicker for the electrode cycled with the E2 electrolyte. This trend agreed with HAXPES data, suggesting that the electrolyte composition greatly impacts the CEI chemistry, with E2 promoting more extensive decomposition products accumulating during cycling.

In positive ion polarity for both cycling states, the SIMS spectra from electrode surfaces are dominated by organic and fluorinated components such as PVdF and LiF along with inorganic components like Li_2_CO_3_ (Figures 7 and 8a,b). As sputtering progresses into the bulk of the electrodes, lithium manganese oxide and metal ions become dominant, implying a transition from the surface CEI components to the electrode’s structural matrix. The negative ion polarity depth profiles reveal a surface enriched in inorganic components such as lithium fluoride and phosphate-containing components, likely derived from electrolyte salt degradation (Figures 7 and 8c,d). Metal fluorides tend to concentrate within the intermediate region between the outermost surface and the bulk electrode.

ToF-SIMS profiles suggest a dual-layer CEI structure, consistent with previous findings.^19^ The outer layer predominantly comprises electrolyte degradation products such as lithium fluoride, lithium carbonate, and phosphorus components. These products evolve due to decomposition of the electrolyte salt and solvents.^10,32,33^ Despite lithium fluoride being the dominant component at the surface, phosphorus compounds also appear due to the reactivity of LiPF_6_ with trace water, generating PF_5_ and acidic species such as HF and POF_3_ (eqs 2 and 3).^23,34^ Water may arise from the oxidative decomposition of carbonate solvents, fueling these reaction further. The inner layer is composed of transition metal fluorides (MF_*x*_), formed by the interaction of transition metal ions with acidic species such as HF.^33,35^ This formation may lead to dissolution products migrating from the cathode to the anode over time.^36^ Such migration contributes to capacity fade by occupying active lithium (Li^+^) sites, thus reducing the overall available of Li^+^ sites.^37^

$$\mathrm{LiP}F_{6}\to \mathrm{LiF}+PF_{5}$$ 1

$$PF_{5}+H_{2}O\to 2\mathrm{HF}+\mathrm{PO}F_{3}$$ 2

$$P{\mathrm{OF}}_{3}+ne^{-}+n{\mathrm{Li}}^{+}\to \mathrm{LiF}+{\mathrm{Li}}_{x}{\mathrm{PO}}_{y}F_{z}$$ 3

Figures 9 and 10 present 3D SIMS images of NMC811 electrodes at the S.C.D. and E.O.L. states, respectively, after cycling in the E1 and E2 electrolytes. In each figure, the top two rows display the spatial distribution of selected positive and negative ions in the electrode cycled with the E1 electrolyte, while the bottom two rows correspond to the E2 electrolyte.

**Figure 9:**
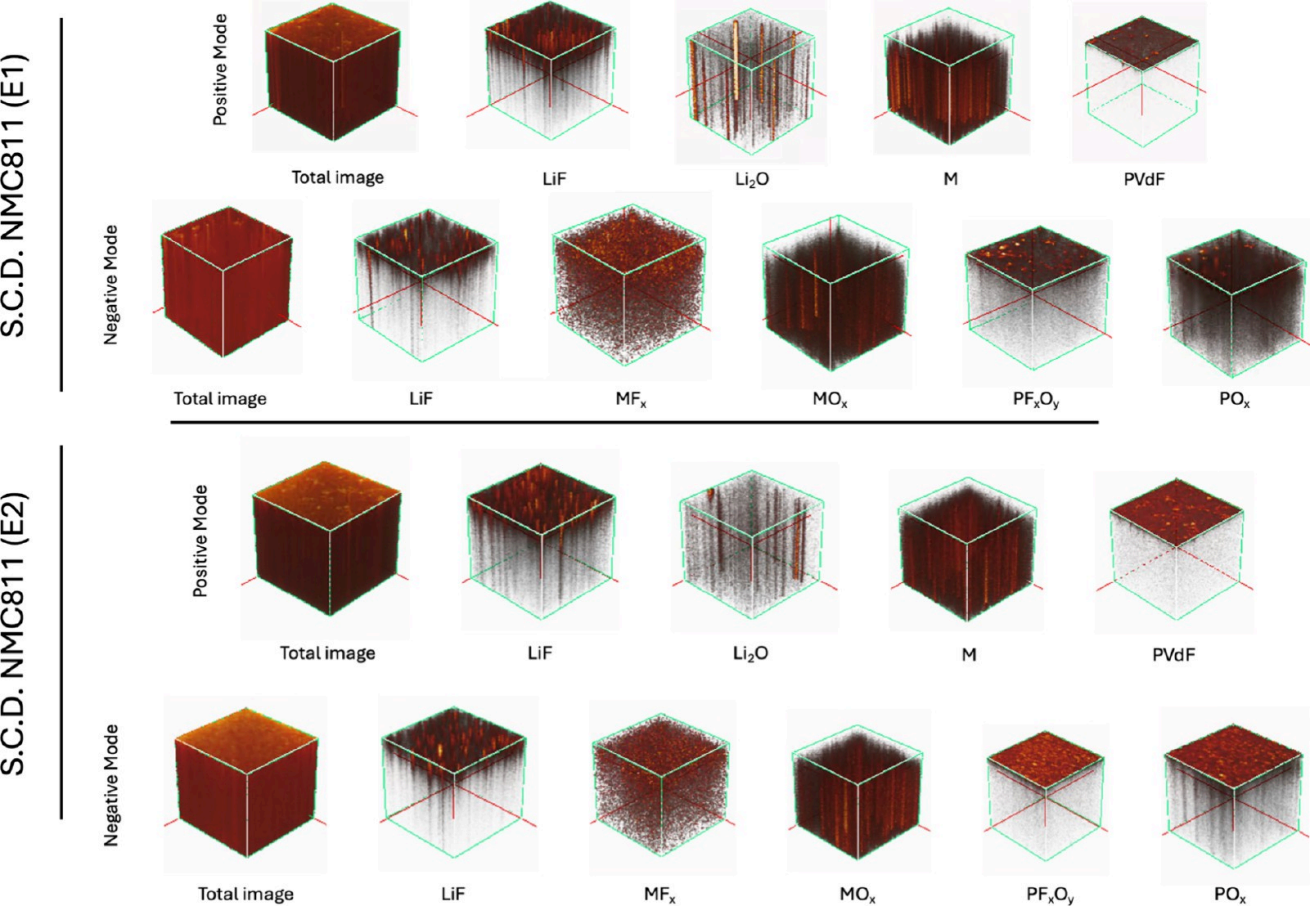
ToF-SIMS 3D images of the S.C.D. NMC811 electrode, showing both negative and positive ion modes. The top two rows depict the positive and negative ion modes for the E1 electrolyte, while the bottom two rows show the corresponding images for the E2 electrolyte.

**Figure 10:**
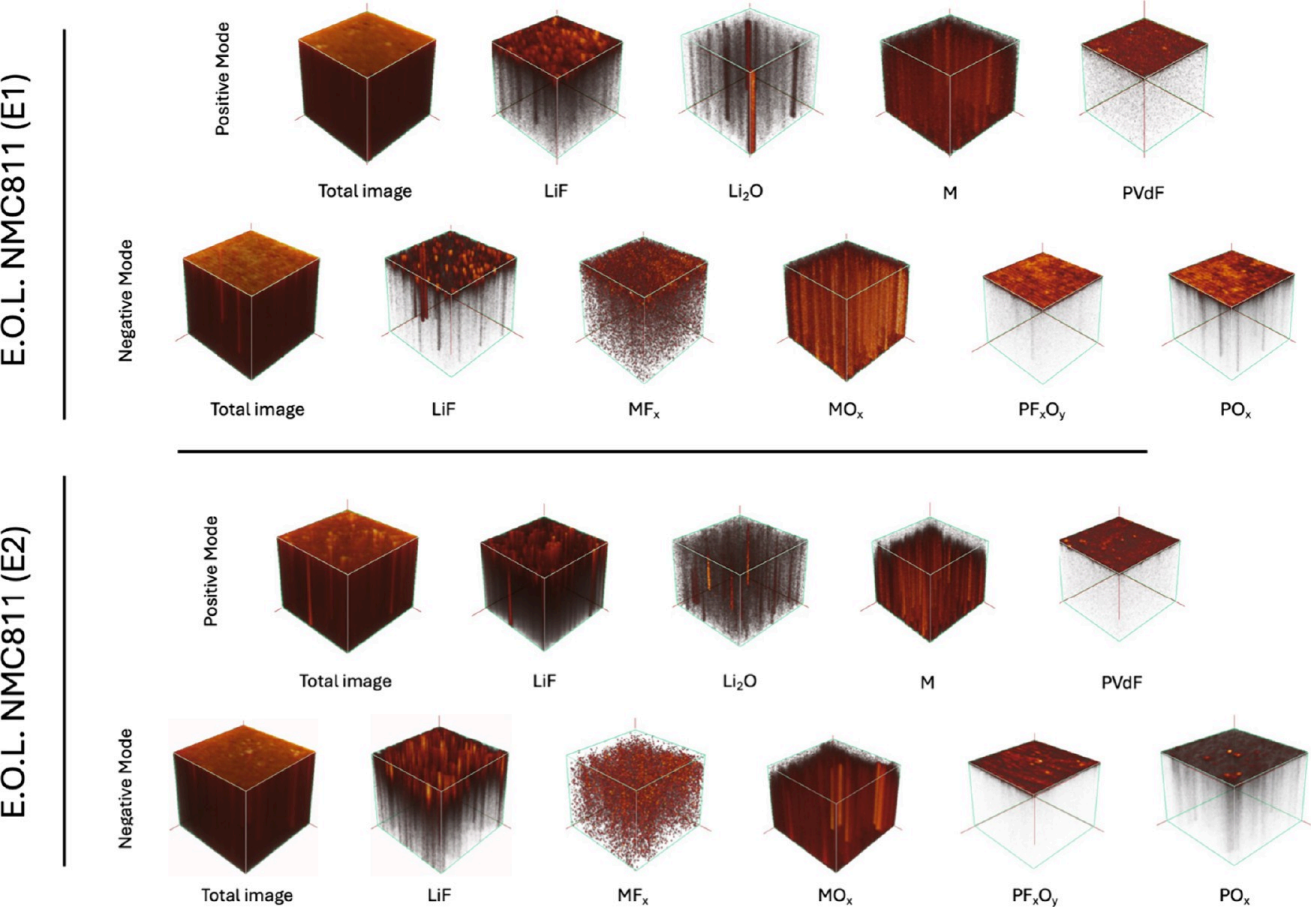
ToF-SIMS 3D images of the E.O.L. NMC811 electrode, showing both negative and positive ion modes. The top two rows depict the positive and negative ion modes for the E1 electrolyte, while the bottom two rows show the corresponding images for the E2 electrolyte.

The representative ions for each component are detailed in Table S4. In the positive ion mode, lithium fluoride is represented by the sum of [Li_2_F]^+^ and [Li_3_F_2_]^+^ ions, transition metal (M) by [Mn]^+^, [Ni]^+^, and [Co]^+^, lithium oxide by [Li_3_O]^+^, and PVdF binder by the [C_3_F_4_H]^+^. In the negative ion mode, lithium fluoride is represented by the [LiF_2_]^−^, phosphorus oxyfluoride by the [PF_2_O_2_]^−^, metal fluorides by the combined [MnF_3_]^−^, [NiF_3_]^−^, and [CoF_3_]^−^ ions, and metal oxides by the sum of [MnO_3_]^−^, [NiO_2_]^−^, and [CoO_2_]^−^ ions. Phosphorus oxides are represented by [PO_2_]^−^ and [PO_3_]^−^ ions.

In both electrolytes for both cycling states, lithium fluoride, PVdF, metal fluorides, phosphorus oxyfluoride, and phosphorus oxide predominantly accumulate at the electrode surface, while lithium oxide, transition metals, and metal oxides dominate the bulk. Notably, PVdF, phosphorus oxyfluoride, and phosphorus oxide consistently localize near the electrode surface across both systems.

At the S.C.D. state, metal fluorides penetrate more in the bulk for both electrolytes. However, at the E.O.L. state, this penetration is significantly deeper in the electrode cycled with E2, indicating a thicker CEI layer. Additionally, at the S.C.D. state, the absence of transition metal and transition metal oxide signals near the surface in both electrolytes suggests a relatively thick CEI layer in both electrolyte systems. However, at the E.O.L. states, these signals remain absent in the E2-cycled electrode, further supporting the observation that the CEI layer formed in E2 is thicker and more extensible. These results indicate that upon cycling, the E2 electrolyte promotes thicker CEI growth compared to E1.

Figures 11 and 12 show ToF-SIMS images of electrode surfaces in positive ion modes for NMC811 in S.C.D. and E.O.L. states cycled in E1 and E2 electrolytes. Both figures illustrate surface distributions of lithium oxide, lithium carbonate, PVdF, lithium fluoride, and nickel metal. In both states, in addition to LiF, the electrodes cycled in E2 are covered with Li_2_O and Li_2_CO_3_, whereas in E1, these components are confined to more localized areas. The colocalization of lithium carbonate and lithium oxide within the same regions (e.g., bottom right corner of Figure 12a) suggests a potential coformation of these components, which may reflect the reactivity of lithium oxide. In addition, lithium oxide, associated with lithium ions, occupies binder-free regions (e.g., top left corner of Figure 11a), suggesting that PVdF may inhibit lithium insertion. This could cause lithium to accumulate in binder-free areas (Figure S3). The isolative properties of PVdF likely hinder lithium transport, limiting movement in the regions it covers.^38,39^

**Figure 11:**
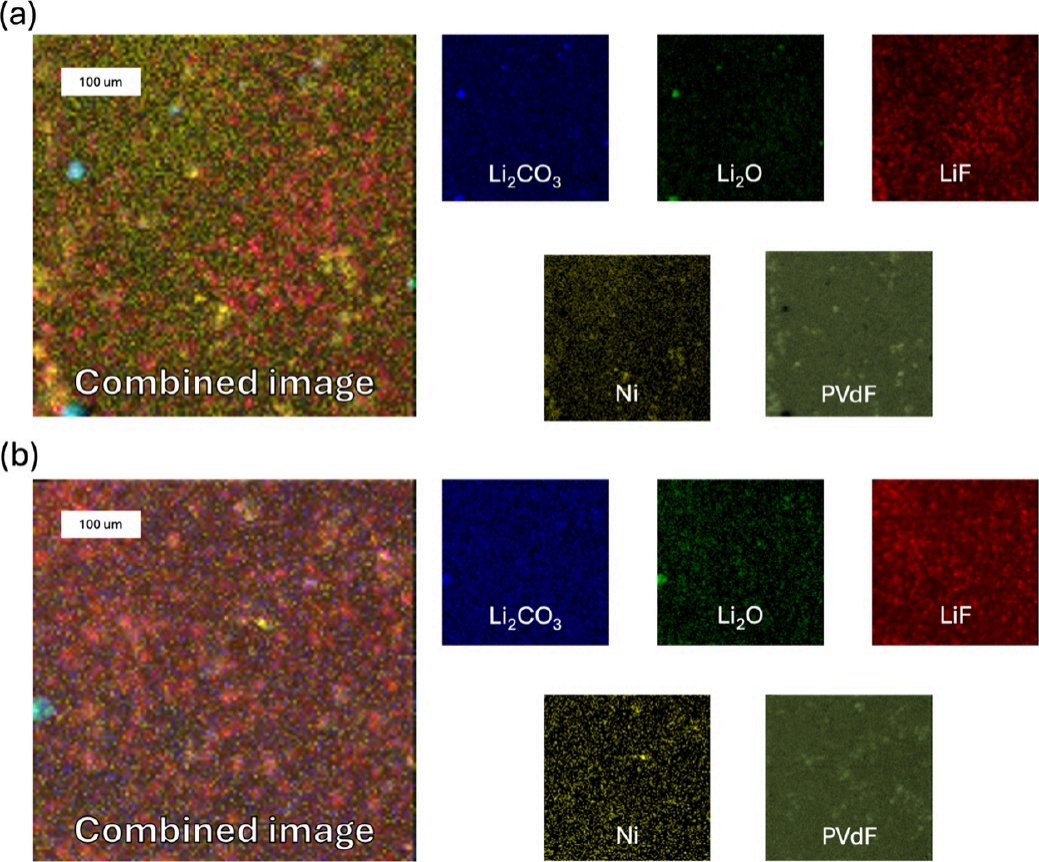
ToF-SIMS surface visualization of the distribution of positive ion components in the S.C.D. state. Shown are the spatial distributions of Li_2_O, Li_*2*_CO_3_, PVdF, LiF, and Mn for electrodes cycled with (a) E1 and (b) E2 electrolytes. Images were acquired over a 500 × 500 μm^2^ area with a resolution of 128 pixels × 128 pixels.

**Figure 12:**
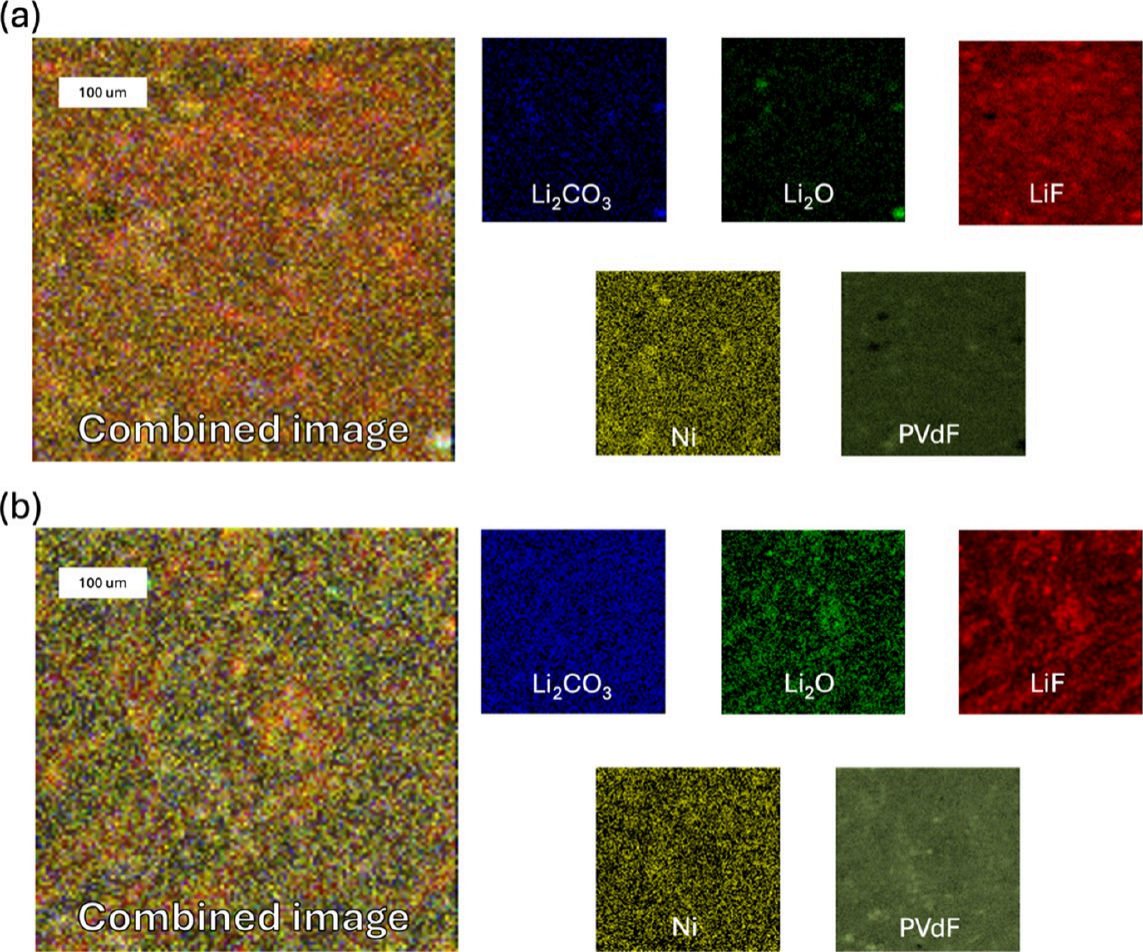
ToF-SIMS surface visualization of the distribution of positive ion components in the E.O.L. state. Shown are the spatial distribution of Li_2_O, Li_*2*_CO_3_, PVdF, LiF, and Mn for electrodes cycled with (a) E1 and (b) E2 electrolytes. Images were acquired over a 500 × 500 μm^2^ area with a resolution of 128 × 128 pixels.

Figures 13 and 14 display ToF-SIMS images of the electrode surfaces in negative ion mode. These illustrate the surface distribution of phosphorus oxide, fluorophosphate, organic carbon, metal oxides, and metal fluorides. In this mode, carbon is relatively uniform across the electrode surface, while phosphorus oxides and fluorophosphate species are colocalized. At the S.C.D. state, these species are more uniformly distributed in E2, while in E1, they appear more localized. At the E.O.L. state, they become less abundant and localized in E2 but are uniformly distributed in E1.

**Figure 13:**
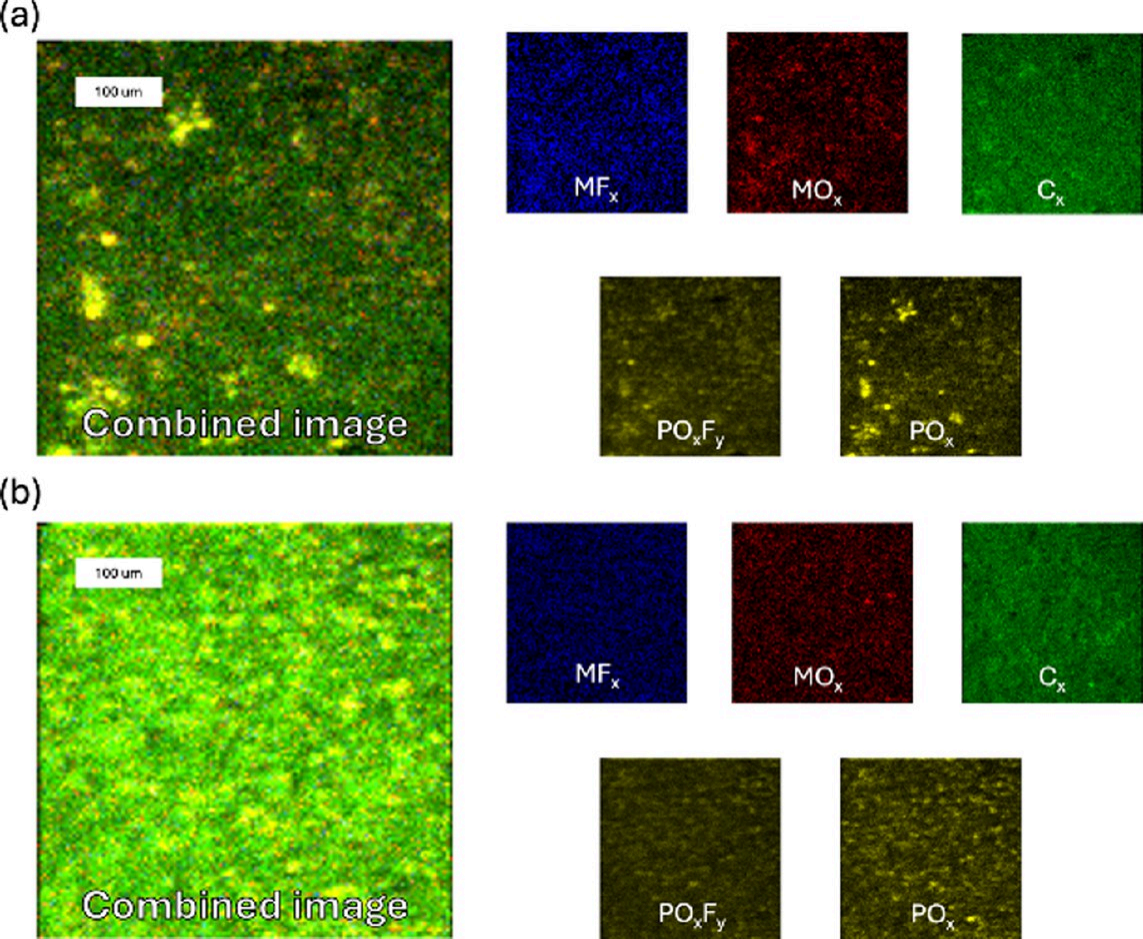
ToF-SIMS surface visualization of the distribution of positive ion components in the S.C.D. state. Shown are the spatial distributions of PO_*x*_F_*y*_, PO_*x*_, MO_*x*_, C_*x*_, and MF_*x*_ for electrodes cycled with (a) E1 and (b) E2 electrolytes. Images were acquired over a 500 × 500 μm^2^ area with a resolution of 128 × 128 pixels.

**Figure 14:**
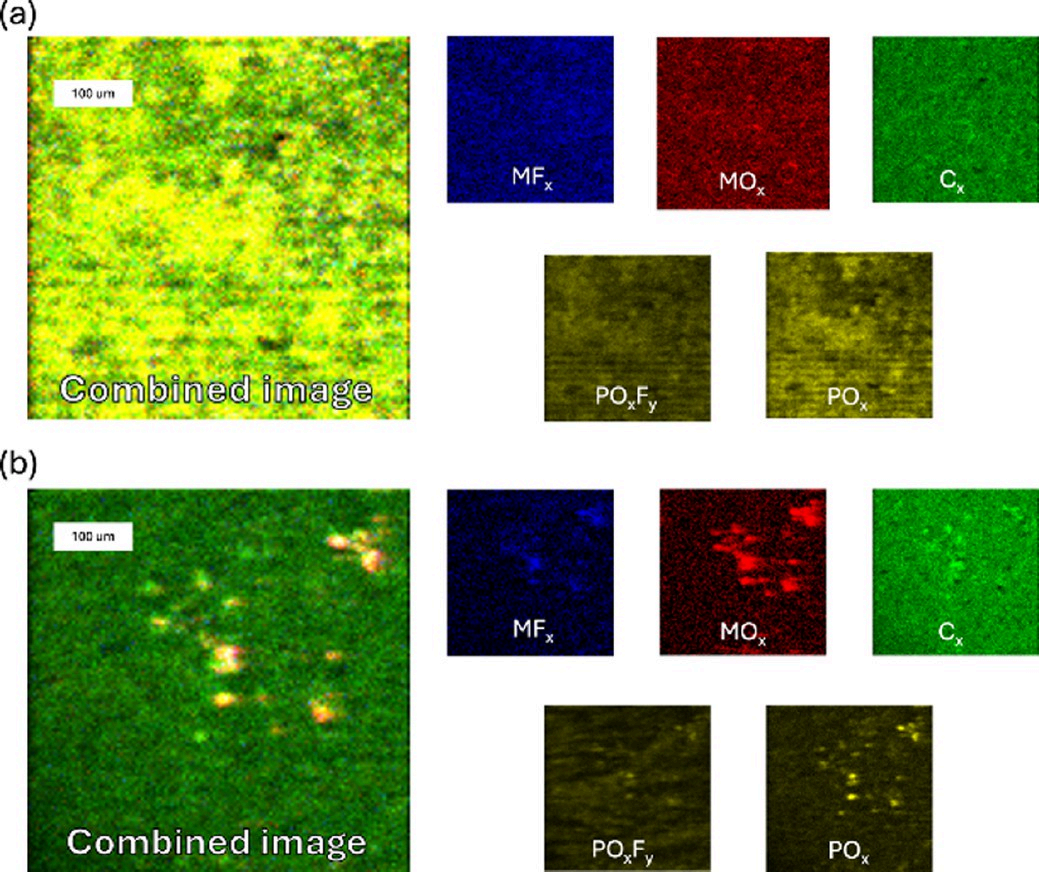
ToF-SIMS surface visualization of the distribution of positive ion components in the E.O.L. state. Shown are the spatial distribution of PO_*x*_F_*y*_, PO_*x*_, MO_*x*_, C_*x*_, and MF_*x*_ for electrodes cycled with (a) E1 and (b) E2 electrolytes. Images were acquired over a 500 × 500 μm^2^ area with a resolution of 128 × 128 pixels.

Notably, phosphorus oxides, fluorophosphates, and metal fluorides colocalize with regions rich in metal oxides, suggesting a spatial correlation between salt decomposition products and the active material. This indicates that electrolyte salt degradation is more noticeable near the electrochemically active sites, likely due to the catalytic effects of the active materials on the decomposition pathways.

Overall, these results suggest that E2 generates a progressively more inorganic-rich LiF-dominated CEI over cycling. This is corroborated by the HAXPES F 1s spectra (Figure 5) and ToF-SIMS surface spectra (Figure 6), which both show significantly greater LiF accumulation at the E.O.L. state in the electrode cycled with E2. Such inorganic components are recognized to enhance the passivation layer, suppress electrolyte–electrode reactivity, and prevent transition metal dissolution. Together, these findings suggest that E2 forms a more robust protective interphase on Ni-rich NMC cathodes, enabling an improved long-term cycling stability.

### 3.3 Systematic Discussion of the CEI Formation Factors

The formation and properties of the CEI layers are governed by multiple factors, as summarized in Table 2. First, the surface of nickel-rich electrodes (e.g.*,* NMC811) is more reactive, catalyzing electrolyte oxidation and leading to a thicker CEI layer compared to lower-nickel content materials.^37c,40^ Second, the state of charge (SoC) and number of cycles significantly influence the CEI chemistry and thickness.^40,41^ Charging to high SoC or extended cycling alters CEI composition. In our study, while the N.C. state forms a negligible CEI layer, the S.C. state shows a noticeable CEI layer. Third, electrolyte composition plays a pivotal role: the addition of DEC in E2 produces an inorganic-rich and more robust CEI layer compared to DEC-free solvent (E1).^42^ Finally, the nonconductive binder (e.g., PVdF) affects Li-ion transport and leads to uneven electrolyte distribution.^38^

**Table 2:** Key Factors Affecting CEI Formation ON NMC Electrodes

factors	influence on CEI formation	manifestation in this study
nickel content	higher Ni-content cathodes are more reactive compared to lower Ni-content, leading to enhanced electrolyte decomposition and thicker CEI formation	comparison of NMC111, NMC 532, and NMC 811show that thicker CEI is associated with higher Ni-content
state of charge (SoC) and cycling	electrolyte decomposition depends on both SoC and cycling history; CEI thickness and composition alter with cycling and voltage	a negligible CEI layer is observed at N.C. states, while the S.C. states exhibit a thicker CEI; for NMC811, cycling with different electrolytes reveals a distinct CEI CEI growth behavior
electrolyte composition	electrolyte composition determines CEI structure, chemistry, and uniformity	in our study, the addition of DEC in E2 produces a more inorganic-rich CEI, distinct from CEI formed in the DEC-free electrolyte (E1)
binder (PVdF)	nonconductive binder such as PVdF impedes Li-ion transport	in our study, PVdF is found to limit Li-ion insertion locally

## 4 Conclusions

This study demonstrates that CEI layer formation on NMC cathodes is chemically driven, forming even without applying bias. However, the CEI layer thickens during charging, as confirmed by comparing N.C. and S.C. states across NMC111, NMC532, and NMC811 compositions. The data also reveal that CEI thickness increases with nickel content in both states, underscoring nickel’s role in accelerating electrolyte decomposition.

Comparative analysis using ToF-SIMS and HAXPES reveals that, for NMC811 cycled in the E1 electrolyte, the CEI layer reaches its maximum thickness in the S.C. state, followed by a gradual decrease in the S.C.D. and E.O.L. states. However, the CEI formed in the E2 electrolyte continues to grow throughout cycling, reaching its maximum thickness in the E.O.L. state. This comparative result shows that E2 leads to more continuous CEI growth and deeper penetration into the electrode, indicating its stronger interaction with the cathode surface.

ToF-SIMS depth profiles and 3D images illustrate a dual-layered CEI layer structure: outer layer composed of electrolyte decomposition products and an inner layer rich in transition metal fluorides. This dual-layer structure indicates a complex degradation pathway that likely impacts lithium-ion transport and cycling stability. Understanding this layered composition helps clarify capacity fade mechanisms and underscores the role of electrolyte stability in prolonging battery life.

These findings suggest that the cycling condition, nickel content, and electrolyte composition significantly influence the CEI growth and composition. A higher nickel ratio aggravates electrolyte decomposition and promotes thicker CEI formation, particularly in electrolytes with higher reactivity, which is consistent with prior hypotheses regarding Ni-induced surface reactivity.^26,43^

Optimizing NMC cathodes thus requires a careful balance of nickel content and electrolyte design to achieve a stable, effective CEI layer and improved long-term cycling performance. Overall, our results emphasize the complementary strengths of ToF-SIMS and HAXPES in probing buried interphases, revealing the value of combining both techniques for a more comprehensive analysis of CEI evolution in LIBs.

## Supplementary Material



## Notes

HAXPES O 1s spectra of pristine NMC811 powder, C 1s spectra of NMC811 electrodes cycled in E1 and E2 electrolytes at various states of charge, visualizations of Li_2_O and PVdF component distributions in the NMC811 electrode at the S.C.D. and E.O.L. states, tables for O 1s and F 1s component assignments, complete lists of mass (*m*/*z*) and mass accuracy (ppm) for both positive and negative ions detected by ToF-SIMS, and list of positive and negative ions displayed in the SIMS 3D images (PDF)

## Abbreviations

LIBs: lithium-ion batteries
N.C.: not-charged
S.C.: single-charged
S.C.D.: single-charged and discharged
E.O.L.: end-of-life
CEI: cathode electrolyte interphase
NMC: lithium nickel manganese cobalt oxide
ToF-SIMS: Time-of-Flight Secondary Ion Mass Spectrometry
HAXPES: Hard X-ray Photoelectron Spectroscopy
