# Supplementary material for: Characterization of Chemical Degradation in Lithium-Ion Batteries Using Secondary Ion Mass Spectrometry (SIMS) and Hard X‑ray Photoelectron Spectroscopy (HAXPES)
Source: ACS Omega. 2025 Nov 19;10(47):57607–22. doi: 10.1021/acsomega.5c08521 (PMC12676502; doi:10.1021/acsomega.5c08521)
Supplement: Supplementary file 1 [file ao5c08521_si_001.pdf]

Supporting information for:

Characterization of Chemical Degradation in Lithium-Ion Batteries Using Secondary Ion Mass Spectrometry (SIMS) and Hard X-ray Photoelectron Spectroscopy (HAXPES)

Abdulrhman H. Alsaedi[1,3], Ben F. Spencer[2], Sadia Sheraz[1], Alex S. Walton[1] and Nick P. Lockyer[1]*

[1]Department of Chemistry and Photon Science Institute, The University of Manchester, Manchester M13 9PL, United Kingdom.

[2]Department of Materials and Henry Royce Institute, The University of Manchester, Manchester M13 9PL, United Kingdom.

[3]Department of Chemistry, faculty of Science, University of Jeddah, Jeddah 23218, Saudi Arabia.

[*] Corresponding Author. Email: Nick.lockyer@manchester.ac.uk

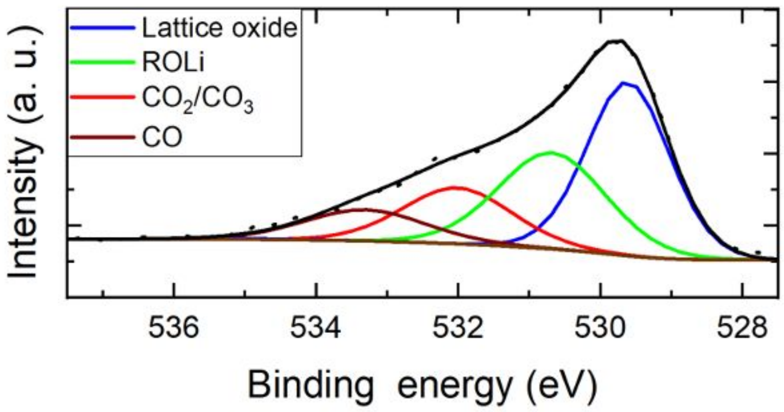

Figure S1. O 1s of pristine NMC811 powder.

* Corresponding Author. Email: Nick.lockyer@manchester.ac.uk

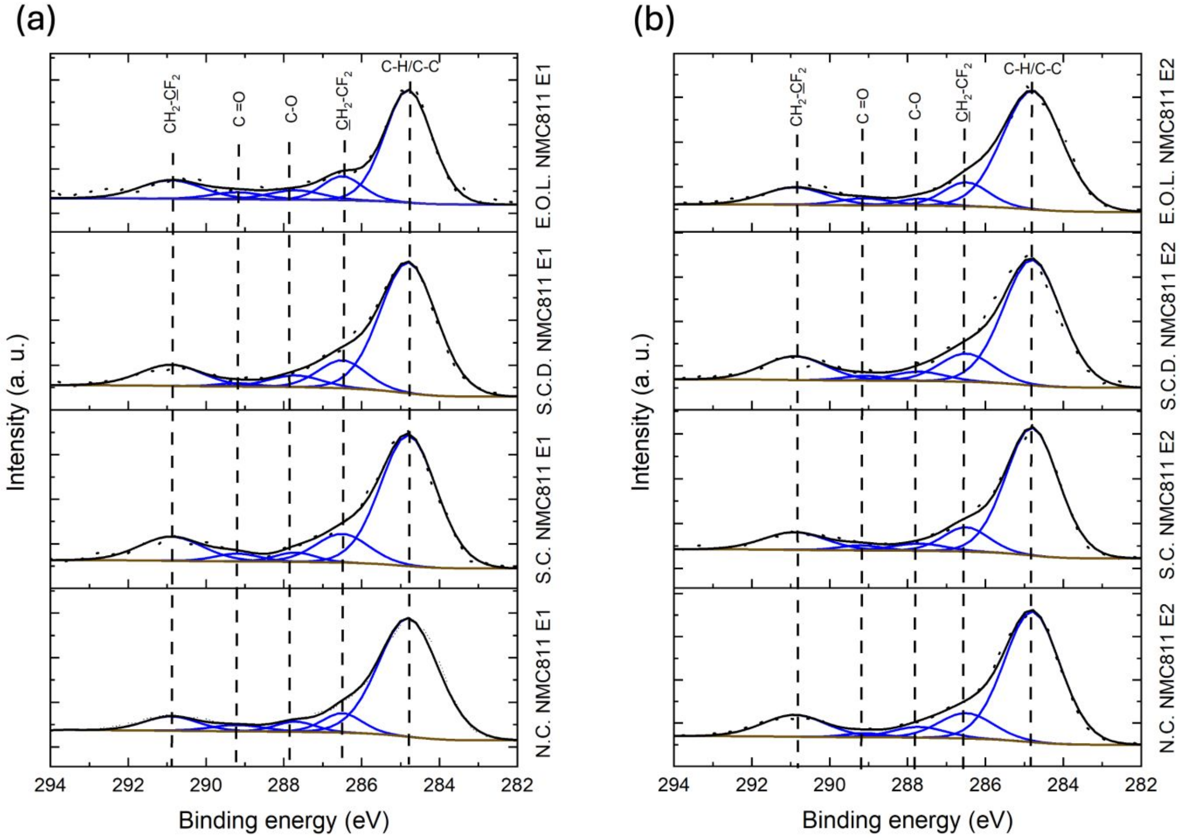

Figure S2. C 1s spectra of the NMC811 electrodes cycled in (a) E1 and (b) E2 electrolyte at different states of charge.

* Corresponding Author. Email: Nick.lockyer@manchester.ac.uk

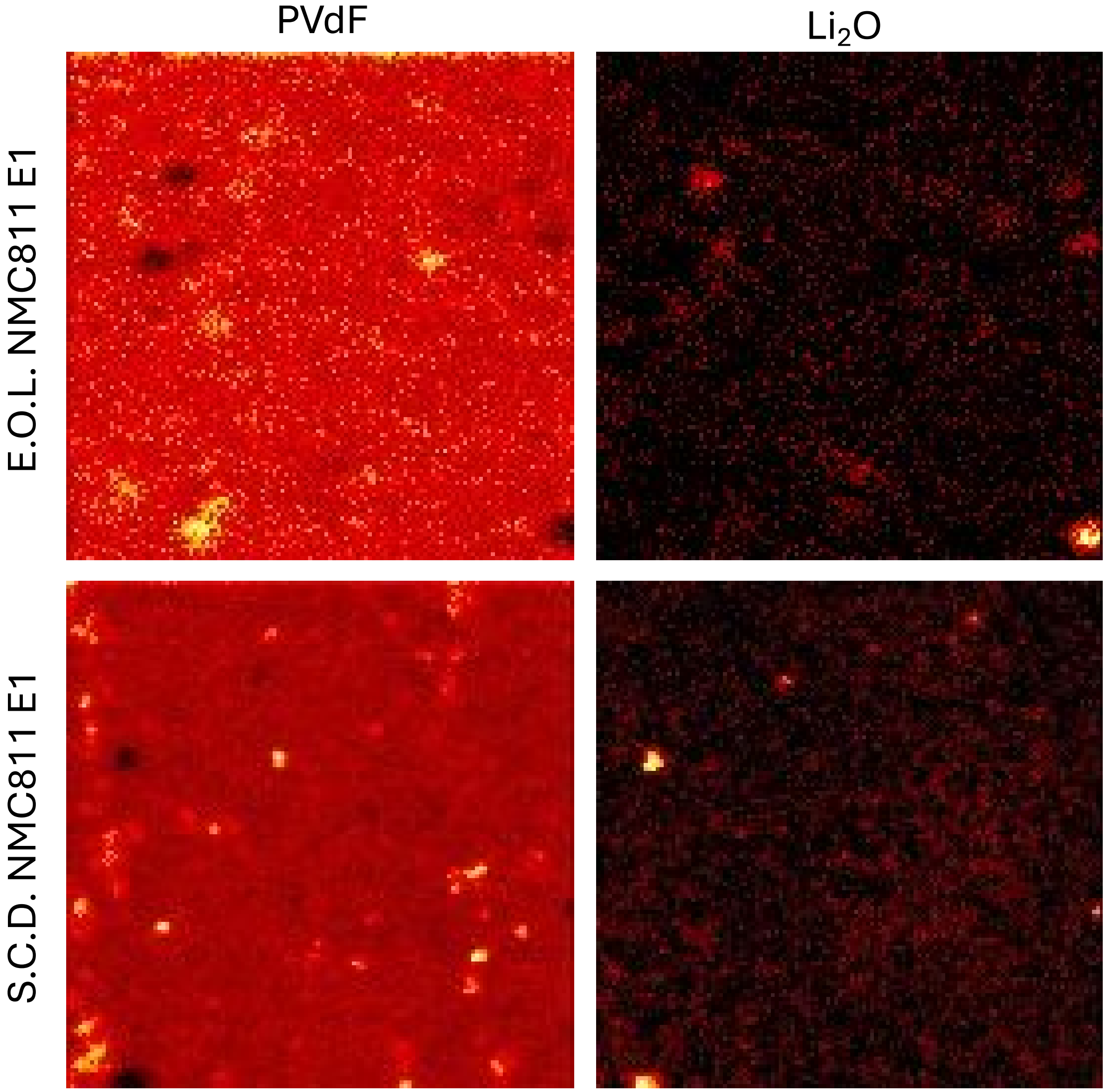

Figure S3. Visualization of Li_2O and PVdF components distribution on E.O.L. of NMC811 electrode in the S.C.D. and E.O.L. states.

* Corresponding Author. Email: Nick.lockyer@manchester.ac.uk

Table S1. Peak assignment for each peak for O 1s and F 1s.

Peaks	Binding energy (eV)	Assignments
O 1s	~529.7 eV	Lattice oxide
	~530.8 eV	ROLi (alkoxide)
	~532.0 eV	CO_2/CO_3 (semicarbonate or carbonate)
	~533.3 eV	CO (esters or polyether)
	~535.0 eV	$Li_xPF_yO_z$
F 1s	~685.0 eV	MF/LiF
	~687.8 eV	PVdF/$Li_xPF_yO_z$
C 1s	~248.8 eV	C-C/C-H
	~286.6 eV	$\underline{C}H_2$-CF_2
	287.8 eV	C-O
	289.2 eV	C=O
	~290.8 eV	CH_2-$\underline{C}F_2$

Table S2. A list of mass (m/z) and mass accuracy (ppm) for the positive ions detected in the ToF-SIMS analysis.

Positive ions	Mass (m/z)	Mass accuracy (ppm)
**$[Li_2F]^+$**	33.0304	0.666
**$[Li_3O]^+$**	37.0430	1.889
**$[Mn]^+$**	54.9379	2.657

* Corresponding Author. Email: Nick.lockyer@manchester.ac.uk

**$[Ni]^+$**	57.9353	0.811
**$[Co]^+$**	58.9330	3.359
**$[C_3F_4H]^+$**	113.0013	1.212

Table S3. A list of mass (m/z) and mass accuracy (ppm) for the negative ions detected in the ToF-SIMS analysis.

Negative Ion	Mass (m/z)	Mass accuracy (ppm)
**$[LiF_2]^-$**	45.0128	0.253
**$[PO_3]^-$**	78.9589	5.677
**$[NiO_2]^-$**	89.9260	9.818
**$[CoO_2]^-$**	90.9235	5.631
**$[MnO_3]^-$**	102.9234	5.917
**$[PF_2O_2]^-$**	100.9609	5.156
**$[MnF_3]^-$**	111.9338	5.578
**$[NiF_3]^-$**	114.9308	2.465
**$[CoF_3]^-$**	115.9288	4.074

Table S4. List of positive and negative ions displayed in the SIMS images, along with their corresponding representative ions.

Components	Positive ions	Components	Negative ions
**LiF**	$[Li_2F]^+$/$[Li_3F_2]^+$	LiF	$[LiF_2]^-$
**Li_2O**	$[Li_3O]^+$	MF_x	$[MnF_3]^-$/$[NiF_3]^-$/$[CoF_3]^-$

* Corresponding Author. Email: Nick.lockyer@manchester.ac.uk

**M**	$[Mn]^+$/$[Co]^+$/$[Ni]^+$	MO_x	$[MnO_3]^-$/$[NiO_2]^-$/$[CoO_2]^-$
**PVdF**	$[C_3F_4H]^+$	PF_xO_y	$[PF_2O_2]^-$
**Li_2CO_3**	$[Li_3CO_3]^+$	PO_x	$[PO_2]^-$ /$[PO_3]^-$

* Corresponding Author. Email: Nick.lockyer@manchester.ac.uk
